# Computational Insights into the Unfolding of a Destabilized Superoxide Dismutase 1 Mutant

**DOI:** 10.3390/biology10121240

**Published:** 2021-11-27

**Authors:** Stepan Timr, Fabio Sterpone

**Affiliations:** 1 Laboratoire de Biochimie Théorique (UPR 9080), CNRS, Université de Paris, 13 Rue Pierre et Marie Curie, 75005 Paris, France; 2 Institut de Biologie Physico-Chimique, Fondation Edmond de Rothschild, PSL Research University, 13 Rue Pierre et Marie Curie, 75005 Paris, France; 3 J. Heyrovsky Institute of Physical Chemistry, Czech Academy of Sciences, Dolejskova 2155/3, 18223 Prague 8, Czech Republic

**Keywords:** superoxide dismutase 1, thermal stability, mutation, misfolding, molecular simulations

## Abstract

**Simple Summary:**

To function correctly, most proteins need to fold into well-defined three-dimensional structures. Destabilization of these structures may not only lead to the loss of function, but also to toxic aggregation and fibril formation. These pathological processes have been linked to a number of neurodegenerative diseases. To prevent such processes, it is important to describe factors causing protein destabilization and identify misfolded structures that are at the origin of the toxic behavior. From the experimental point of view, in many cases, it is useful to construct protein models to better investigate the issues of stability, misfolding, and aggregation. Here, indeed, we focus on a mutant model of superoxide dismutase 1, a protein implicated in amyotrophic lateral sclerosis. We apply a state-of-the-art molecular simulation method to verify whether the current computational machinery is able to describe the features of the biochemical model. Namely, our paper provides a microscopic insight into the unfolding of the superoxide dismutase 1 model while highlighting the strengths and limitations of the computational approach. Overall, our investigation opens the route to the computational study of pathological mutants of the superoxide dismutase 1 protein.

**Abstract:**

In this work, we investigate the β-barrel of superoxide dismutase 1 (SOD1) in a mutated form, the isoleucine 35 to alanine (I35A) mutant, commonly used as a model system to decipher the role of the full-length apoSOD1 protein in amyotrophic lateral sclerosis (ALS). It is known from experiments that the mutation reduces the stability of the SOD1 barrel and makes it largely unfolded in the cell at 37 degrees Celsius. We deploy state-of-the-art computational machinery to examine the thermal destabilization of the I35A mutant by comparing two widely used force fields, Amber a99SB-disp and CHARMM36m. We find that only the latter force field, when combined with the Replica Exchange with Solute Scaling (REST2) approach, reproduces semi-quantitatively the experimentally observed shift in the melting between the original and the mutated SOD1 barrel. In addition, we analyze the unfolding process and the conformational landscape of the mutant, finding these largely similar to those of the wildtype. Nevertheless, we detect an increased presence of partially misfolded states at ambient temperatures. These states, featuring conformational changes in the region of the β-strands β4−β6, might provide a pathway for nonnative aggregation.

## 1. Introduction

The majority of proteins adopt a well-defined three-dimensional structure, which is critical for their physiological function [1,2]. Destabilization of the native conformation, potentially followed by toxic aggregation and fibril formation, has been linked to a number of diseases [2,3].

Cu, Zn superoxide dismutase (SOD1) is a cytosolic antioxidant enzyme protecting the cell from oxidative damage caused by oxygen free radicals. Misfolding and toxic aggregation of numerous SOD1 mutants have been linked to the familial form of amyotrophic lateral sclerosis (ALS) [3,4], and the role of SOD1 in the sporadic form of ALS is also debated [3]. In contrast to many other proteins involved in late-onset neurodegenerative diseases, mature SOD1 is highly stable, forming a homodimer containing Cu and Zn cations [5]. Exactly how the diverse set of ALS-related amino-acid mutations promotes SOD1 misfolding and aggregation remains an open question [5]. While a unifying picture is still missing, an increasing amount of evidence points to a mechanism involving the formation of disulfide-reduced apoSOD1 monomers, lacking the two metal ions. The metal depletion as well as the absence of the disulfide bond make these monomers less stable and more likely to misfold and form toxic oligomers [6,7,8].

**Figure 1:**
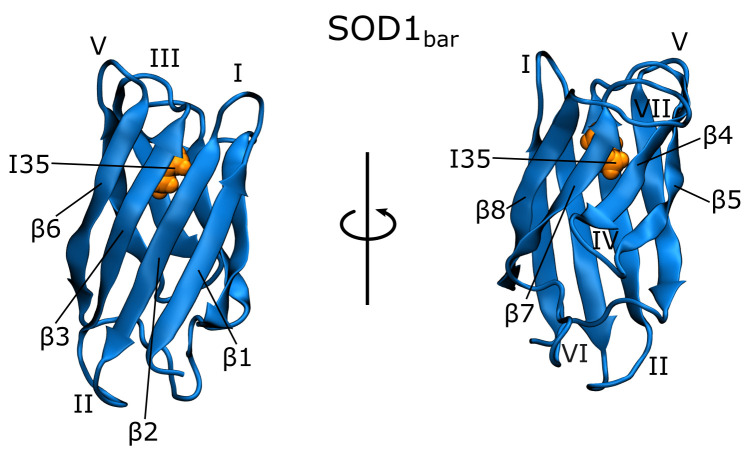
Structure of the SOD1${}_{\mathrm{bar}}$ construct, a metal-free monomeric variant of Cu, Zn superoxide dismutase [13]. Shown are its β-strands β1–β8 and loops I–VII, as well as isoleucine 35 (I35).

Since the crowded cellular environment can have a significant effect on protein stability, recent experiments explored the modulation of SOD1 folding states by the intracellular environment [9,10]. Furthermore, the impact of misfolded SOD1 association on the formation and properties of in vivo biomolecular condensates has been investigated [11]. As an experimental model to study the in vivo localization and misfolding behavior of apoSOD1 monomers, a simplified monomeric variant of the protein (SOD1${}_{\mathrm{bar}}$) is frequently used in these studies [10,12,13,14] (see Figure 1). This variant features two truncated loops (the metal-binding loop IV and the electrostatic loop VII), and it lacks cysteine moieties forming a disulfide bond in the mature SOD1. These changes with respect to the full-length SOD1 give rise to a metal-free monomeric form of the β-barrel which retains the main folding characteristics of the full-length monomer while being well-suited for in-cell NMR and fluorescence assays [9,10,12,13,15].

The loop truncation entropically stabilizes SOD1${}_{\mathrm{bar}}$ relative to the full-length disulfide-reduced apoSOD1 monomer [12]. To bring the stability of SOD1${}_{\mathrm{bar}}$ closer to that of the disulfide-reduced apoSOD1 monomer, the isoleucine 35 to alanine (I35A) mutation is often introduced to the barrel [9,16,17], substantially decreasing the thermal stability of SOD1${}_{\mathrm{bar}}$ and causing a large part of SOD1${}_{\mathrm{bar}}$ molecules to be unfolded in the cell at 37 degrees Celsius [9]. Thus, although the I35A mutation is currently not known to cause familial ALS, the SOD1${}_{\mathrm{bar}}^{I35A}$ mutant constitutes a convenient model system to investigate the effects of the cellular environment on the folding state of the destabilized apoSOD1 monomer.

Misfolding of SOD1 has been studied by means of molecular simulations, mostly performed at a coarse-grained resolution [6,18,19,20,21,22,23,24,25]. We previously explored the unfolding of SOD1${}_{\mathrm{bar}}$ and its interactions in a crowded environment by performing coarse-grained and atomistic molecular dynamics (MD) simulations [10,26]. The simulations allowed us to characterize interactions of the β-barrel with protein crowders as a function of its conformation and predict its most fragile region susceptible to thermal unfolding.

In this paper, we explore the thermal unfolding of SOD1${}_{\mathrm{bar}}^{I35A}$ and compare it with that of “wildtype” SOD1${}_{\mathrm{bar}}$ (SOD1${}_{\mathrm{bar}}^{\mathrm{WT}}$). To this end, we perform extensive atomistic MD simulations using the Replica Exchange with Solute Scaling (REST2) scheme [27,28]. We examine whether the REST2 method in combination with Amber a99SB-disp [29] and CHARMM36m [30], that is, two recent protein force fields optimized for simulation of folded and disordered proteins, succeeds in capturing the destabilization stemming from the I35A mutation. Furthermore, we analyze in detail the unfolding of SOD1${}_{\mathrm{bar}}^{I35A}$ and inquire into its conformational states to see if the I35A mutation substantially affects the folding landscape of the SOD1${}_{\mathrm{bar}}^{\mathrm{WT}}$ construct, known to match the folding behavior of the full-length apoSOD1 monomer.

## 2. Materials and Methods

### 2.1. REST2 Simulations

Protein misfolding can occur on the time scale of many years [31]. Despite the rapid progress in computer power, the sampling of such rare and slow events in direct MD trajectories remains out of reach, motivating the development of enhanced-sampling approaches. REST2 [27,28] is a variant of replica exchange molecular dynamics (REMD) [32], a method that significantly accelerates the crossing of energy barriers—and thus also the exploration of protein conformational landscapes—by simulating multiple replicas of the system at increased temperatures while allowing for exchanges of geometries between the temperatures. Compared to REMD, REST2 uses a Hamiltonian-rescaling procedure to selectively heat protein degrees of freedom, which results in a substantial reduction in the number of replicas required to sample the conformational landscape of the given system [27]. We demonstrated previously the effectiveness of REST2 at reproducing thermal stability trends for small and midsize proteins in dilute- [28,33] and crowded conditions [26,34,35,36].

In this paper, all-atom REST2 simulations [27,28] were performed using the GROMACS 2019.4 software [37] patched with the Plumed 2.5.3 package [38]. Two distinct sets of force field parameters were used to describe the protein: Amber a99SB-disp [29] and CHARMM36m [30]. The a99SB-disp protein force field was coupled with the a99SB-disp water model, whereas CHARMM36m was combined with the TIP3P water model [39]. In both cases, K${}^{+}$ and Cl${}^{-}$ ions were described with the default parameters for the respective force field. A 1.2 nm cutoff was applied to short-range non-bonded interactions. In addition, van der Waals forces were smoothly switched to zero between 1.0 and 1.2 nm in the CHARMM36m simulations. Long-range electrostatic interactions were evaluated using the particle mesh Ewald method [40]. To improve the computational efficiency of the simulation by removing the fastest degrees of freedom, the LINCS algorithm [41] was used to constrain the lengths of all protein bonds involving hydrogen, and the SETTLE algorithm [42] was employed to keep water molecules rigid. The temperature of the system was maintained at 300 K by the velocity rescaling thermostat with a stochastic term [43], which was coupled separately to the proteins and to the rest of the system with a time constant of 1 ps.

The initial SOD1${}_{\mathrm{bar}}$ structures were prepared using the following procedure. The crystal structure (PDB ID 4BCZ [13] for SOD1${}_{\mathrm{bar}}^{\mathrm{WT}}$ and PDB ID 4XCR [9] for SOD1${}_{\mathrm{bar}}^{I35A}$) was processed with the GROMACS pdb2gmx tool [37], using the default choices for the protonation states of the amino acid residues. The histidine 43 residue was modeled with a hydrogen atom in the δ-position while the other histidines had a hydrogen atom in the ϵ-position. Subsequently, the protein was placed in a ∼7.5 nm cubic box, and solvated by ∼13,000 water molecules and a 150 mM concentration of K${}^{+}$ and Cl${}^{-}$ ions, with three additional K${}^{+}$ ions to neutralize the net charge of the simulation box. This system then underwent a short energy minimization which was followed by a six-step relaxation protocol (see Table S1 for more details). The first two short simulations, which were performed in the NVT ensemble, were followed by four NpT trajectories simulated at a pressure of 1.01 bar, maintained by the Berendsen barostat [44] with the time constant set to 1 ps. Harmonic restraints were applied to all heavy atoms of the protein, where different force constants, gradually decreasing to zero, were employed to restrain the heavy atoms of the backbone and the side chains of the protein (Table S1). The final geometry resulting from the equilibration protocol formed the starting point for the REST2 simulation.

**Figure 2:**
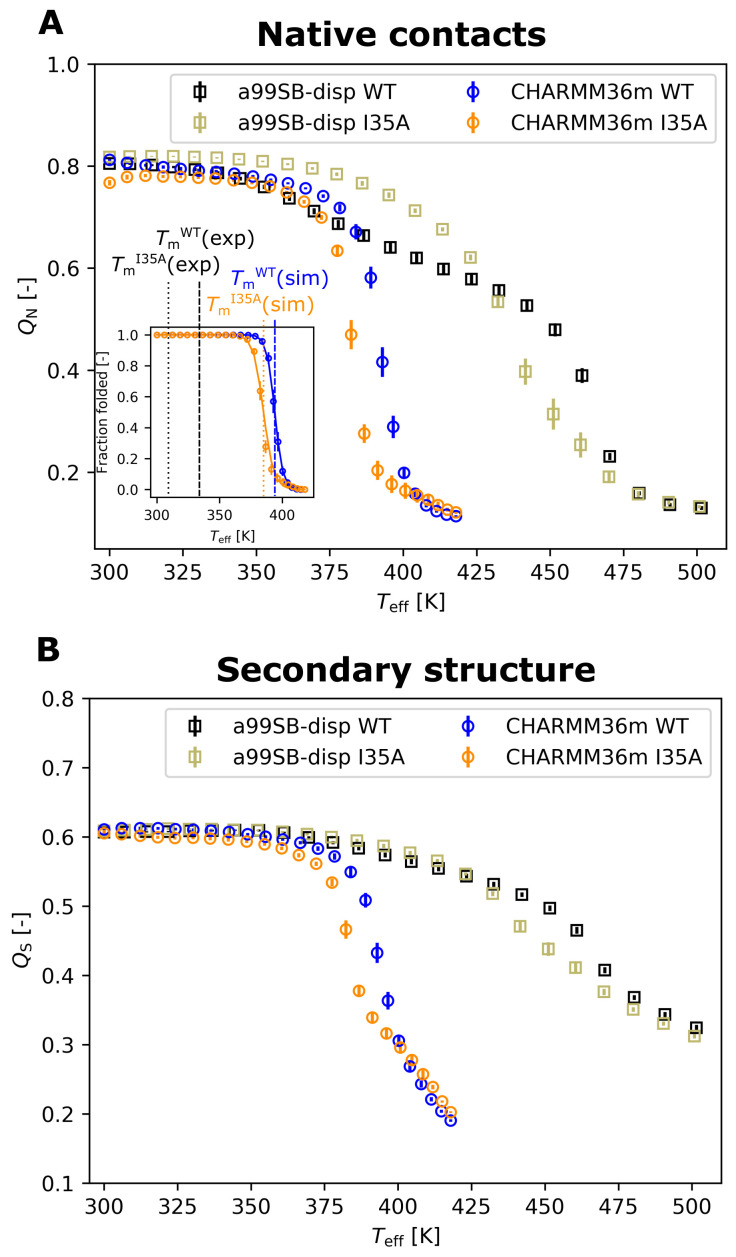
Shifts in the thermal stability of SOD1${}_{\mathrm{bar}}$ due to the isoleucine 35 to alanine (I35A) mutation in the a99SB-disp [29] and CHARMM36m [30] force fields. (**A**) Fraction of native contacts ($Q_{N}$) as a function of the effective temperature $T_{\mathrm{eff}}$ for SOD1${}_{\mathrm{bar}}^{\mathrm{WT}}$ and SOD1${}_{\mathrm{bar}}^{I35A}$. The inset shows the temperature-dependent fraction of folded geometries for SOD1${}_{\mathrm{bar}}^{\mathrm{WT}}$ and SOD1${}_{\mathrm{bar}}^{I35A}$ described by the CHARMM36m force field. The fraction of folded geometries was obtained from $Q_{N}$ by assuming a two-state model; see Supplementary Methods for more details. The dashed and dotted vertical lines in the inset indicate the melting temperatures of SOD1${}_{\mathrm{bar}}^{\mathrm{WT}}$ and SOD1${}_{\mathrm{bar}}^{I35A}$, respectively, obtained from the CHARMM36m simulation (colored lines) and determined experimentally in dilute conditions [9] (black lines). (**B**) Secondary structure content ($Q_{S}$), i.e., the fraction of SOD1${}_{\mathrm{bar}}$ residues found in an α-helix, β-sheet, β-bridge, or a turn, evaluated by the GROMACS do_dssp tool and plotted as a function of $T_{\mathrm{eff}}$.

In the Hamiltonian rescaling procedure of the REST2 approach, the protein was treated as the “solute” while the rest of the system formed the “solvent”. The solute temperatures ranged from 300 K to 698 K (Table S2), and exchanges of replicas were attempted every 5 ps. The temperature spacing ensured sufficiently high average exchange probabilities(>0.15) for neighboring pairs of solute temperatures. In the REST2 simulations, the pressure was maintained at 1.01 bar using the Parrinello-Rahman barostat [45] with a time constant of 1 ps, and the system was propagated using the leap-frog algorithm [46] with a time step of 2 fs. The lengths of the a99SB-disp REST2 simulations reached 1.5 μs, while the CHARMM36m REST2 simulations were 1 μs long. Using a mean-field approach introduced in Ref. [28], we calculated an “effective temperature” ($T_{\mathrm{eff}}$), approximating the temperature that was experienced by the protein for each rescaled Hamiltonian. The first 400 ns of each REST2 simulation were omitted from the analysis. The statistical convergence of the structural observables extracted from the trajectory, namely the fraction of native contacts and the secondary structure content (see Supplementary Methods and the caption of Figure 2 for more details), was evaluated with a block scheme by dividing the trajectory into 50 ns intervals and calculating the average separately for each interval. From these averages, we calculated the standard error of the mean of the given observable.

### 2.2. Alchemical Calculations

To estimate the change in the free energy of unfolding due to the I35A mutation, we performed alchemical calculations utilizing the free-energy code of GROMACS with the same force fields and simulation parameters as above (except the fact that all bonds were constrained) and with the soft-core α and σ parameters set to 0.3 and 0.25, respectively. From the respective REST2 simulation of SOD1${}_{\mathrm{bar}}^{\mathrm{WT}}$, we extracted 10 folded and 10 unfolded geometries, which were sampled in 50 ns time intervals at $T_{0}$ and $T_{20}$, respectively (see Table S2). For CHARMM36m, only protein geometries were extracted and were subsequently rehydrated using the same equilibration protocol, as described above. Hybrid I35A geometries were then prepared using the mutate.py script from the pmx package (version 2.0) [47,48]. For each hybrid geometry, a 100 ps transition from the non-mutated (A) to the mutated (B) state was simulated, and a 10 ns equilibration simulation was performed for each hybrid geometry in state A and a 20 ns equilibration simulation in state B. From the last 10 ns of each equilibration trajectory, we extracted 50 snapshots in uniform intervals. For each of these snapshots, we performed a 100 ps transition to the opposite state (B and A, respectively). The derivatives, recorded during the transitions, of the Hamiltonian with respect to the coupling parameter λ were used by the analyze_dhdl.py script from the pmx package to calculate the free energy of mutation using the Crooks Gaussian Intersection method for each of the 10 folded and 10 unfolded geometries.

## 3. Results

### 3.1. Thermal Stability from REST2 Simulations

Experimentally, the I35A mutation was found to strongly destabilize SOD1${}_{\mathrm{bar}}$, lowering the protein’s melting temperature by 25 degrees Celsius to 35.6 degrees Celsius [9]. As a consequence, the majority of SOD1${}_{\mathrm{bar}}^{I35A}$ molecules are expected to be unfolded at the human body temperature.

We examined whether the REST2 approach [27,28] in combination with two state-of-the-art protein force fields, a99SB-disp [29] and CHARMM36m [30], captured the destabilizing effect of the mutation. REST2 was shown previously to be successful at capturing stability differences between mesophilic and thermophilic variants of the same protein [33] as well as at evaluating the effects of molecular crowding on the thermal stability of proteins [26,35]. Destabilization stemming from a single-residue replacement which, moreover, has almost no impact on the structure of the native state [9] constitutes a challenging test of the sensitivity of the approach to small local modifications of the amino-acid sequence. We found that only CHARMM36m correctly reproduced the destabilization due to the I35A mutation, even if the shift in the melting temperature was somewhat underestimated (−9 degrees Celsius; see Figure 2). In contrast, the a99SB-disp force field failed to consistently reproduce the destabilizing effect, which was only present at high temperatures. At lower temperatures, the mutant even appeared to be stabilized with respect to the wildtype (see Figure 2). The a99SB-disp force field also exhibited a higher residual secondary structure content at high temperatures (see Figure 2B).

It should be noted that both force fields significantly overestimated the melting temperatures (see Figure 2), a phenomenon that we previously saw in REST2 simulations of a protein in dilute [33] and crowded solutions [26,35], as well as in powder [34,36] and that has commonly been reported for protein force fields [49]. This upshift in the observed melting temperature was particularly marked for a99SB-disp. As a consequence, the REST2 simulations predicted that both SOD1${}_{\mathrm{bar}}^{\mathrm{WT}}$ and SOD1${}_{\mathrm{bar}}^{I35A}$ are folded at 37 degrees Celsius.

The stability curves obtained with the a99SB-disp force field converged more slowly than those with CHARMM36m (see Figure S1), suggesting a slower unfolding kinetics of SOD1${}_{\mathrm{bar}}$ when described with the a99SB-disp force field compared to CHARMM36m. Moreover, the wildtype Amber simulation exhibited a strong presence of partially unfolded intermediate states at high temperatures (Figure S2), whereas fully unfolded states tended to be more populated for the mutant at such temperatures (Figure S3).

### 3.2. Energetics of the Mutation

To explain why the two force fields performed differently in capturing the mutation-induced destabilization, we analyzed more closely the energetics of the mutation, as described by each force field. For CHARMM36m, featuring two rather well separated populations of folded and unfolded states (see Figures S4 and S5), we were able to construct two-state models and derive stability curves from them (see Figure S6). According to these stability curves, the I35A mutation caused a destabilization at 37 degrees Celsius by $\Delta \Delta G_{u}\left(37\;{}^{\circ}C\right)=-15$ kJ/mol. This value is consistent with those reported from NMR experiments (−19.2 kJ/mol) [9] and fluorescence stability assays (−12.5 kJ/mol) [10] at 37 degrees Celsius. For the a99SB-disp force field, we were unable derive the two-state stability curves, owing to the presence of multiple intermediate states (see Figures S2 and S3). Therefore, to compare the two force fields, we turned to alchemical free-energy calculations. Specifically, for each force field, we extracted ten folded and ten unfolded geometries from the respective REST2 simulation of SOD1${}_{\mathrm{bar}}^{\mathrm{WT}}$ and subjected them to an alchemical free-energy transformation (see Section 2 and Figure S7 for details) to obtain an estimate of the change in the unfolding free energy $\Delta G_{u}$ upon the I35A mutation. We found that, despite the different outcomes of the REST2 simulations, the alchemical calculations yielded similar results for the two force fields, namely a net destabilization by −11±3 kJ/mol for CHARMM36m and −12±2 kJ/mol for a99SB-disp due to the I35A mutation. Again, these values compare rather favorably with the experimentally reported destabilization. The reason for the different results obtained for a99SB-disp from REST2 and from alchemical free energy perturbation will be discussed later on in the paper. Previous computational analysis of an isoleucine-to-alanine mutation in barnase [50] showed that major contributions to the destabilization arose from bonding terms involving degrees of freedom of the mutated side chain and from non-bonded interactions of that side chain with its environment in the folded protein, whereas hydration effects were reported to play only a minor role [50]. Nevertheless, our alchemical calculations revealed that the destabilizing effect of the I35A mutation does correlate with the number of contacts of the I35 side chain with water in the unfolded state (see Figure S8).

### 3.3. Effect of the I35A Mutation on the Folding Landscape

**Figure 3:**
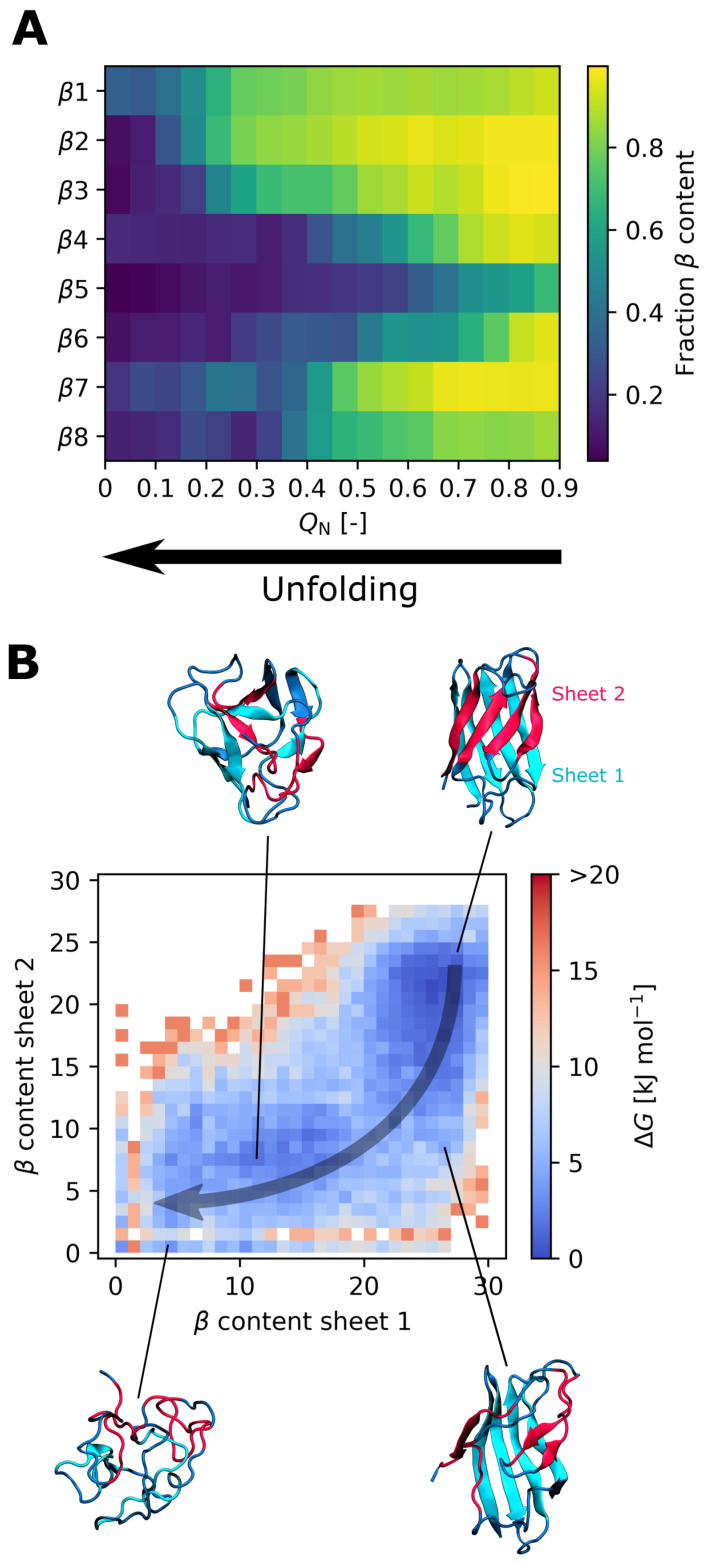
Unfolding of SOD1${}_{\mathrm{bar}}^{I35A}$ as described by the CHARMM36m force field. (**A**) Overall histogram of the β-sheet secondary-structure content in individual β-strands of SOD1${}_{\mathrm{bar}}^{I35A}$ as a function of the native contact fraction $Q_{N}$. The values are normalized with respect to the crystal structure. (**B**) Free-energy landscapes of SOD1${}_{\mathrm{bar}}^{I35A}$ unfolding near the melting temperature ($T_{\mathrm{eff}}=$387 K). The two collective variables correspond to the number of residues with the β-sheet secondary structure in the β-sheet 1 (formed by the β-strands β1, β2, β3 and β6) and the β-sheet 2 (β4, β5, β7 and β8). The arrow illustrates a possible path from the folded state to the unfolded state.

**Figure 4:**
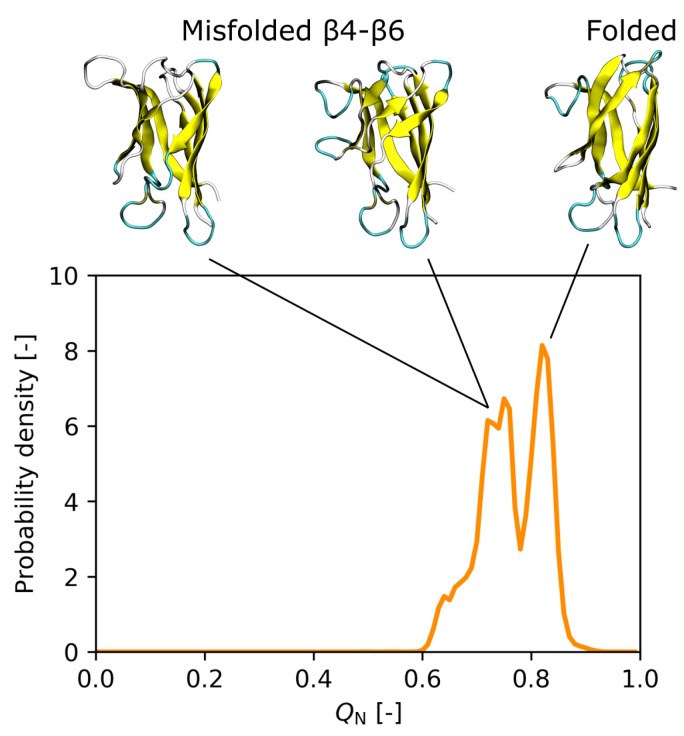
Histogram of the native contact fraction $Q_{N}$ for SOD1${}_{\mathrm{bar}}^{I35A}$ at 300 K obtained with the CHARMM36m force field. The SOD1${}_{\mathrm{bar}}^{I35A}$ mutant features an increased population of misfolded conformations at the ambient temperature, which are mainly characterized by conformational changes in the β4–β6 region.

To predict the effect of the I35A mutation on the SOD1${}_{\mathrm{bar}}$ folding landscape, we focus on the results obtained with the CHARMM36m force field, as it better captured the destabilization caused by the mutation. First, our simulations predict that the unfolding of SOD1${}_{\mathrm{bar}}^{I35A}$ starts in the region of the β-strands β5 and β6, followed by β4 (see Figure 3A). This region coincides with the weak spot that we observed for SOD1${}_{\mathrm{bar}}^{\mathrm{WT}}$ (see Figure S9). Second, the β-sheet 2, consisting of the β-strands β4, β5, β7, and β8 (see Figure 3B), is predicted to be more fragile than the β-sheet 1 (Figure 3B) and typically unfolds before the latter. Again, this is consistent with the unfolding behavior of SOD1${}_{\mathrm{bar}}^{\mathrm{WT}}$ (see Figure S10). Third, compared to SOD1${}_{\mathrm{bar}}^{\mathrm{WT}}$, the SOD1${}_{\mathrm{bar}}^{I35A}$ mutant features an increased population of semi-unfolded intermediate states at ambient temperatures, exhibiting a partially unfolded/misfolded region of β4–β6 (see Figure 4). This rearrangement might be promoted by decreased contacts of nonpolar residues F45, V57, V67, and A65, whose side chains are located on the interior surface of β4–β6, with the side chain of the mutated residue.

## 4. Discussion

The free-energy differences that we obtained from alchemical calculations using the two force fields upon the I35A mutation suggest that the poor performance of a99SB-disp is not caused by a deficiency in its description of the mutation’s energetics. Instead, the failure of a99SB-disp to capture the destabilization of SOD1${}_{\mathrm{bar}}$ could be related to a slower kinetics of unfolding, manifested by an increased presence of semi-unfolded intermediate states and significantly slower convergence of the REST2 simulations compared to CHARMM36m. As a result, the populations of the unfolded state at different temperatures, and the effect of the mutation upon them, could not be sampled correctly. REST2 is a powerful enhanced-sampling simulation technique; however, thorough sampling of protein conformational landscapes is a daunting task. To correctly estimate the stability curve, enough geometries (replicas) must be able to unfold and diffuse along the temperature axis within the simulation time. Moreover, once a replica becomes unfolded, it is unlikely to re-fold fully within the microsecond simulation time for a protein sized similarly to SOD1${}_{\mathrm{bar}}$. This poses clear constraints on the ability of REST2 to sample protein folding equilibria. However, as evidenced by the present results obtained with the CHARMM36m force field, given a favorable unfolding kinetics, REST2 may still capture relative differences in the stabilities of two protein variants, as well as the main characteristics of their unfolding. This makes the REST2 approach useful for identifying misfolded states of SOD1 and other proteins involved in neurodegenerative diseases.

We observed that SOD1${}_{\mathrm{bar}}^{I35A}$ retains the principal characteristics of the SOD1${}_{\mathrm{bar}}^{\mathrm{WT}}$ folding landscape, namely the weak spot formed by the β-strands β5 and β6, initiating thermal unfolding, and the generally more flexible β-sheet 2 (formed by the β-strands β4, β5, β7, and β8) compared to the β-sheet 1. The results of these new CHARMM36m simulations are also consistent with our previous study [26] of SOD1${}_{\mathrm{bar}}^{\mathrm{WT}}$ unfolding in crowded conditions, where we used the Amber ff99SB*-ildn force field [51,52,53] to describe the barrel. Moreover, the β-sheet 2 was described to be more dynamic than the β-sheet 1 in previous experimental and computational studies of apoSOD1 [21,24,54,55]. Thus, our present findings confirm that SOD1${}_{\mathrm{bar}}^{I35A}$, despite carrying a mutation strongly affecting the folding equilibrium, remains a good model system for studying “wildtype” SOD1 unfolding.

Compared to SOD1${}_{\mathrm{bar}}^{\mathrm{WT}}$, the SOD1${}_{\mathrm{bar}}^{I35A}$ mutant accentuates the presence of partially misfolded states at ambient temperatures in the REST2 simulations, with conformational changes occurring mainly in the β4−β6 region. The cleft between β5 and β6 was reported to become partially unstructured in excited states of certain destabilized mutants of the full-length apoSOD1 monomer, which might potentially lead to nonnative oligomerization [7]. In addition, some of these conformations might be related to a compact excited state of SOD1${}_{\mathrm{bar}}$ which was detected by NMR experiments [13] and found to be stabilized by transient interactions with a protein crowder, leading to a slow-down of SOD1${}_{\mathrm{bar}}$ aggregation [16].

Finally, let us note that the global folding and unfolding kinetics of both SOD1${}_{\mathrm{bar}}$ and the full-length apoSOD1 was found experimentally to be largely consistent with a two-state model [9,13,56] although more recent single-molecule experiments detected multiple intermediate states for apoSOD1 [57]. In this sense, the results achieved by CHARMM36m are more consistent with the experimental picture than those obtained with a99SB-disp, which strongly overemphasizes the intermediate-state populations (see Figures S2–S5). Overall, our results point to a more rugged folding landscape in a99SB-disp compared to CHARMM36m.

## 5. Conclusions

In this work, by performing enhanced-sampling MD simulations, we investigated the thermal unfolding and the destabilization of the SOD1${}_{\mathrm{bar}}^{I35A}$ mutant. While this study highlighted certain limitations of the REST2 approach, we found that, in connection with the CHARMM36m force field, the REST2 simulation reproduced the destabilization at least semi-quantitatively. In general, the I35A mutant retained the main features of the in silico WT unfolding; therefore, we conclude that it constitutes a good model system to study the unfolding behavior of apoSOD1 monomers. In addition, the detailed insights into SOD1${}_{\mathrm{bar}}^{I35A}$ unfolding presented in this article may provide valuable information for studies employing SOD1${}_{\mathrm{bar}}^{I35A}$ to explore the role of mutation in aggregation and partitioning into liquid biomolecular condensates [14,58].

## Data Availability

The REST2 trajectories and the derived datasets presented in this study are openly available in the Zenodo repository at dx.doi.org/10.5281/zenodo.5570754.

## Supplementary Materials

The following are available online at https://www.mdpi.com/article/10.3390/biology10121240/s1, Supplementary Methods: Definition of the native contact fraction and Obtaining a stability curve from a two-state model, Figure S1: Convergence of the fraction $Q_{N}$ of SOD1${}_{\mathrm{bar}}^{\mathrm{WT}}$ and SOD1${}_{\mathrm{bar}}^{I35A}$ native contacts in REST2 simulations, Figures S2–S5: Temperature-dependent distributions of the fraction $Q_{N}$ of SOD1${}_{\mathrm{bar}}^{\mathrm{WT}}$ and SOD1${}_{\mathrm{bar}}^{I35A}$ native contacts sampled with a99SB-disp/CHARMM36m, Figure S6: Stability curves obtained from a two-state model for theCHARMM36m REST2 simulations, Figure S7: Free-energy difference arising from the alchemical transformation of isoleucine 35 to alanine, Figure S8: Correlation between the number of water contacts of the I35 side chain and the free-energy differences arising from the I35A alchemical transformation, Figure S9: Overall histogram of the β-sheet secondary-structure content in individual β-strands of SOD1${}_{\mathrm{bar}}^{\mathrm{WT}}$ as a function of the native contact fraction $Q_{N}$ and obtained with the CHARMM36m force field, Figure S10: Free-energy landscapes of SOD1${}_{\mathrm{bar}}^{\mathrm{WT}}$ unfolding at different temperatures of the CHARMM36m REST2 simulation, Table S1: Relaxation protocol used to obtain the initial protein geometry, Table S2: Solute temperatures used in the REST2 simulations.
