# Supplementary material for: Computational Insights into the Unfolding of a Destabilized Superoxide Dismutase 1 Mutant
Source: Biology (Basel). 2021 Nov 27;10(12):1240. doi: 10.3390/biology10121240 (PMC8698278; doi:10.3390/biology10121240)
Supplement: Supplementary file 1 [file biology-10-01240-s001.zip › biology-1448109-supplementary.pdf]

Supplementary Information to: Computational Insights into the Unfolding of a Destabilized Superoxide Dismutase 1 Mutant

Stepan Timr[1,2,3,*], Fabio Sterpone[1,2,*],
[1]Laboratoire de Biochimie Théorique (UPR 9080), CNRS, Université de Paris
[2]Institut de Biologie Physico-Chimique, Fondation Edmond de Rothschild, PSL Research University
[3]J. Heyrovsky Institute of Physical Chemistry, Czech Academy of Sciences

*Correspondence: stepan.timr@jh-inst.cas.cz, fabio.sterpone@ibpc.fr

Supplementary Methods

Definition of the native contact fraction

A pair (i, j) of $\mathrm{SOD1_{bar}}$ Cα carbon atoms i and j separated by at least three amino acid residues in the peptide sequence was considered a native contact if positioned at a distance d_{ij}^{ref} lower than 10 Å in the crystal structure. The fraction of native contacts for a given $\mathrm{SOD1_{bar}}$ conformation was then calculated as

$$Q_{\mathrm{N}} = \frac{1}{N_{\mathrm{ref}}} \sum_{(i,j)} \exp\left[-0.5\left(\max(0, d_{ij} - d_{ij}^{\mathrm{ref}})\right)^2\right] \quad (1)$$

where the sum runs over all the native contacts in the crystal structure, N_{ref} is the number of these native contacts, and d_{ij} is the distance of the atom pair in the given conformation.

Obtaining a stability curve from a two-state model

As the CHARMM36m REST2 simulations exhibited two relatively well-separated populations of folded and unfolded states (see Figures S4–S5), it was possible to set a threshold value $Q_{\mathrm{N}} = 0.4$ separating native-like conformations from denaturated ones in order to calculate the temperature-dependent fraction $r(T)$ of folded states. The free energy of unfolding, that is, the difference in the free energies of the folded- and unfolded states in the two-state model, equaled:

$$\Delta G_{\mathrm{u}}(T) = k_{\mathrm{B}} T \ln \frac{r(T)}{1 - r(T)} \tag{2}$$

The $\Delta G_{\mathrm{u}}(T)$ was fit with the modified Gibbs-Helmholtz equation:

$$\Delta G_{\mathrm{u}}(T) = -\Delta C_{\mathrm{p}} \left[T \left(\ln \left(\frac{T}{T_{\mathrm{m}}} \right) - 1 \right) + T_{\mathrm{m}} \right] + \Delta H_{\mathrm{m}} \left(1 - \frac{T}{T_{\mathrm{m}}} \right) \tag{3}$$

where T_{m} stands for the melting temperature, ΔH_{m} expresses the melting enthalpy, and ΔC_{p} is the specific heat of unfolding. Given a limited statistics in regions where r closely approached 1 or 0, we only included such temperatures in the fit for which $r(T) < 0.99$ and $r(T) > 0.01$.

Supplementary Figures

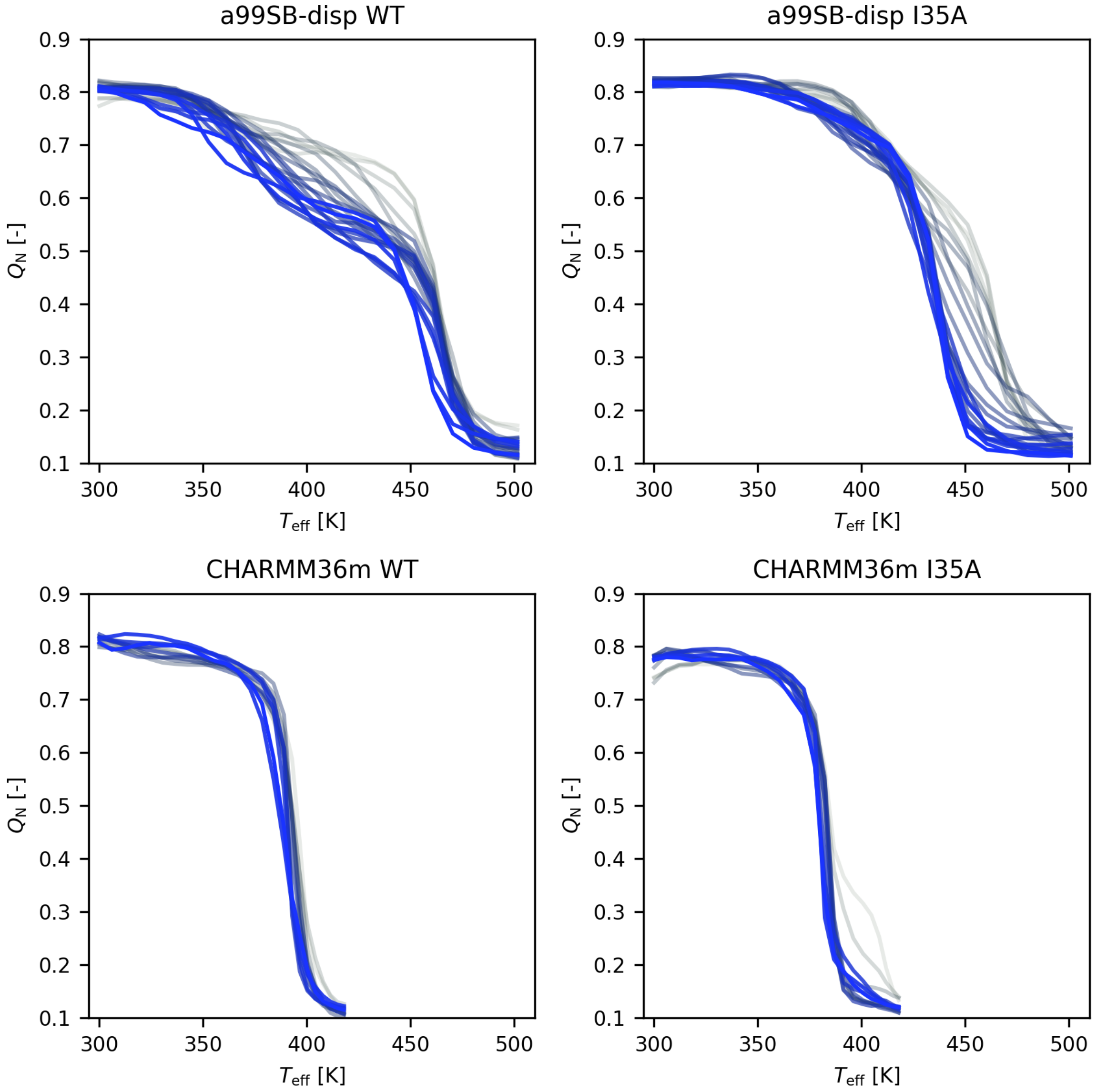

Figure S1: Convergence of the fraction Q_N of $SOD1_{bar}^{WT}$ and $SOD1_{bar}^{I35A}$ native contacts in REST2 simulations. The individual curves were obtained from 50 ns blocks spanning (light gray to blue) the 400−1500 ns interval for a99SB-disp and the 400−1000 ns interval for CHARMM36m.

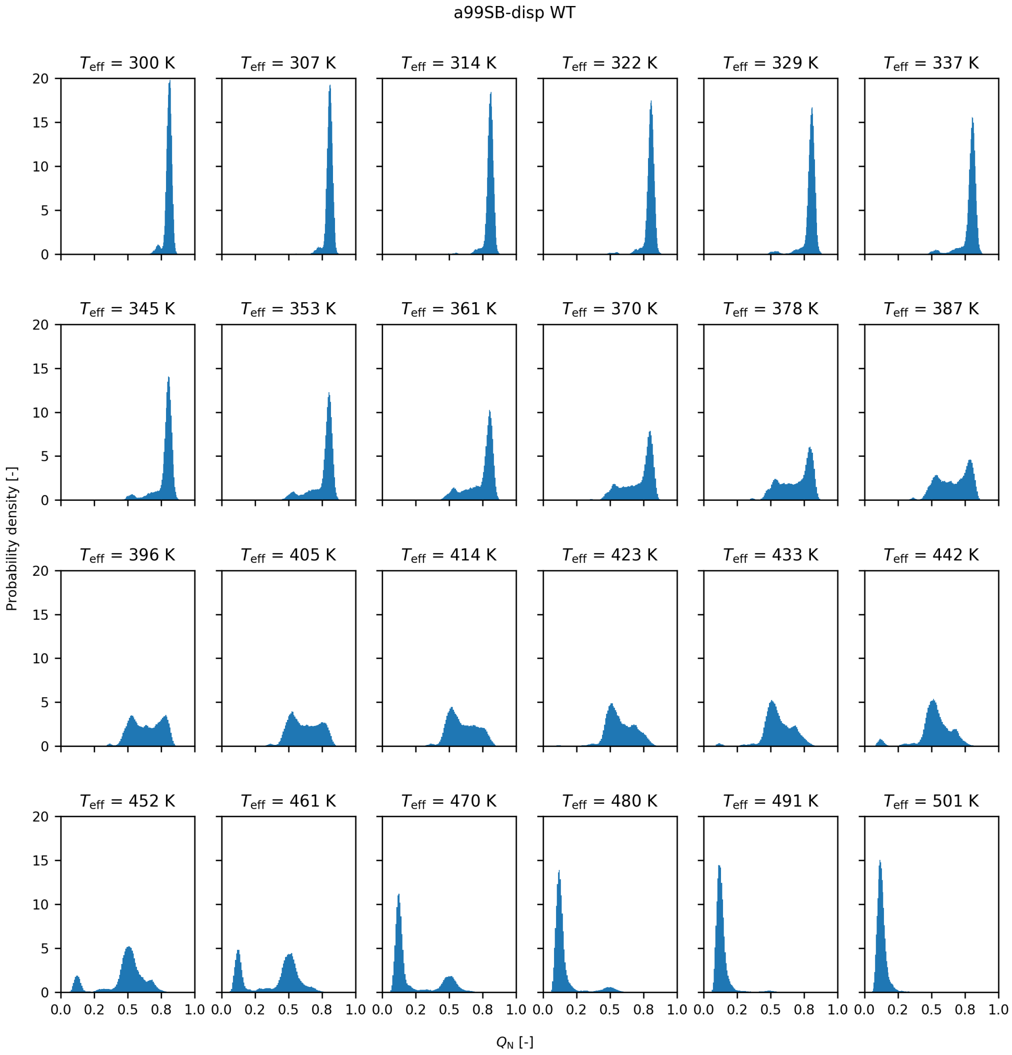

Figure S2: Temperature-dependent distributions of the fraction Q_{N} of $\mathrm{SOD1}^{\mathrm{WT}}_{\mathrm{bar}}$ native contacts sampled with a99SB-disp.

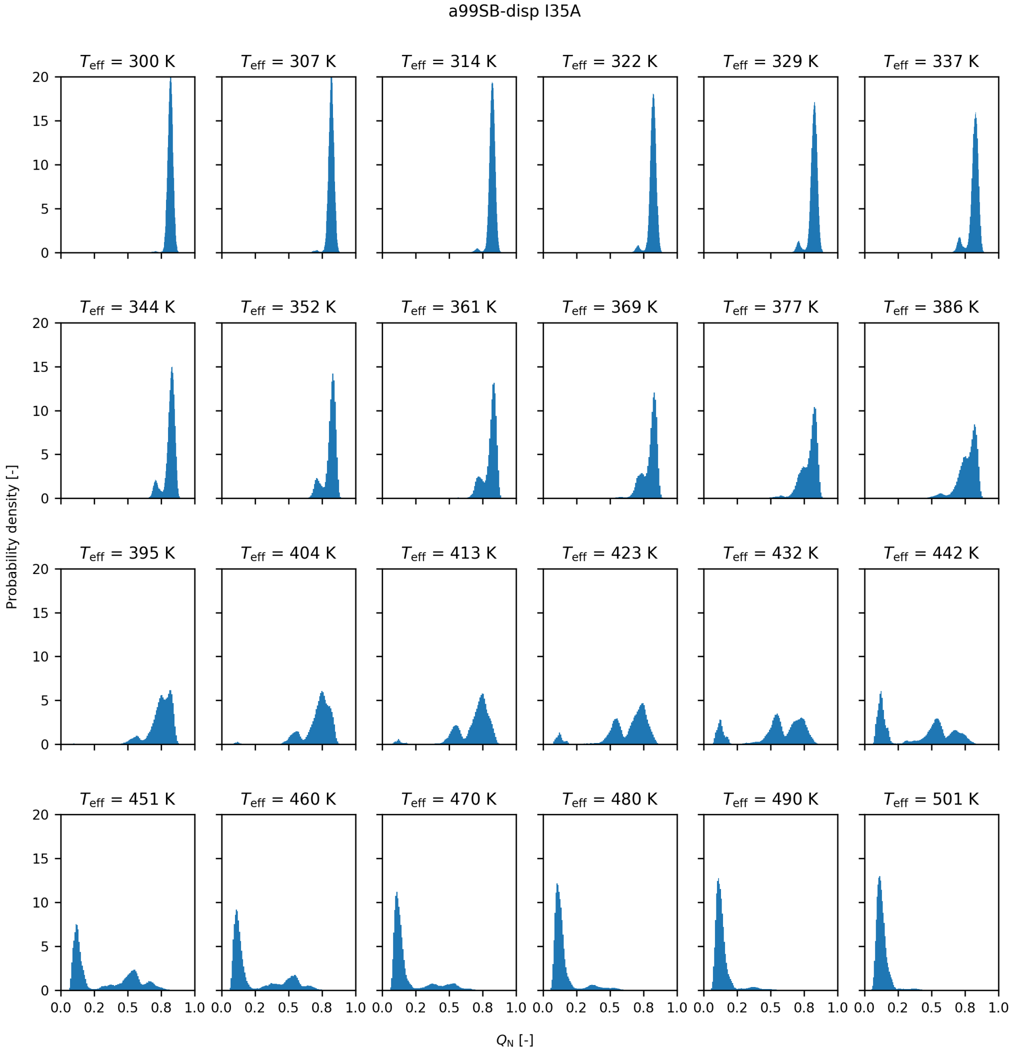

Figure S3: Temperature-dependent distributions of the fraction Q_{N} of $\mathrm{SOD1}^{\mathrm{I35A}}_{\mathrm{bar}}$ native contacts sampled with a99SB-disp.

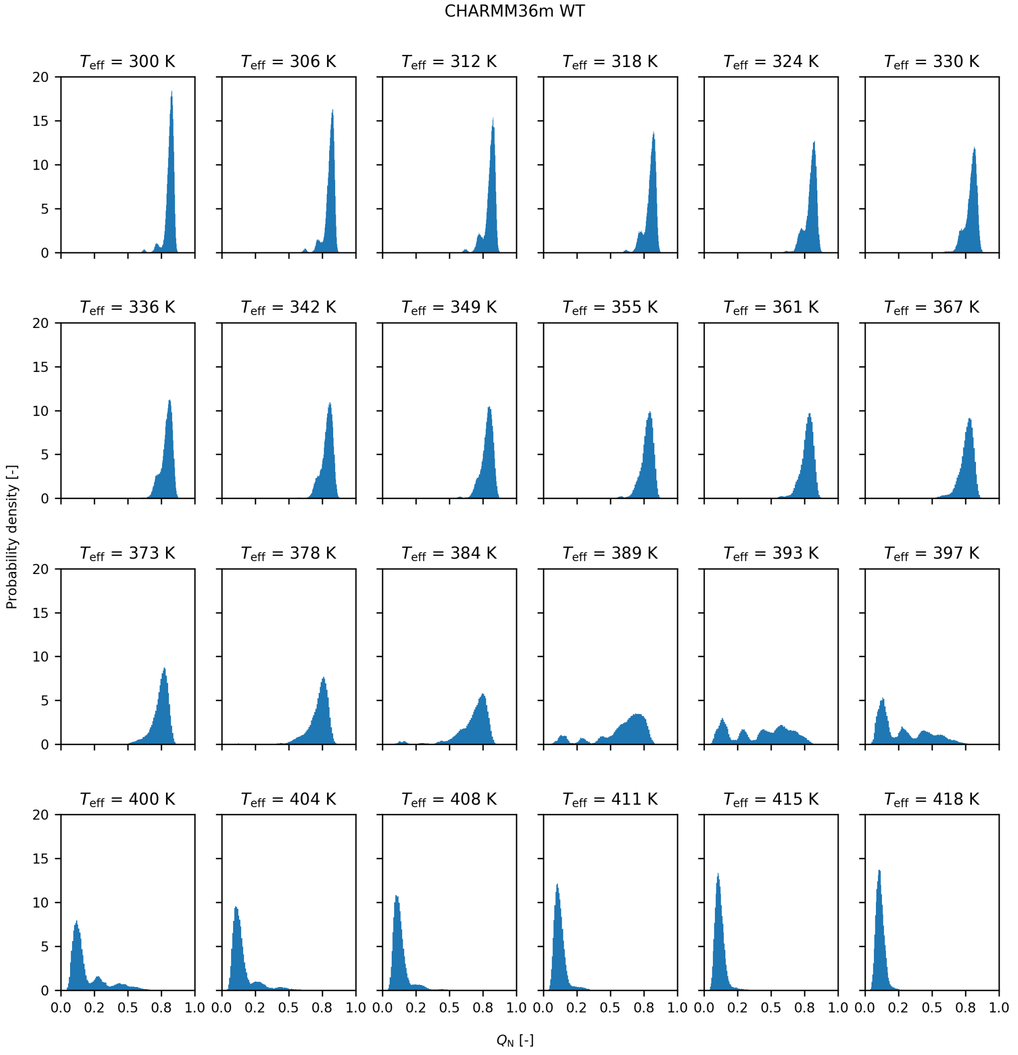

Figure S4: Temperature-dependent distributions of the fraction Q_N of $SOD1^{WT}_{bar}$ native contacts sampled with CHARMM36m.

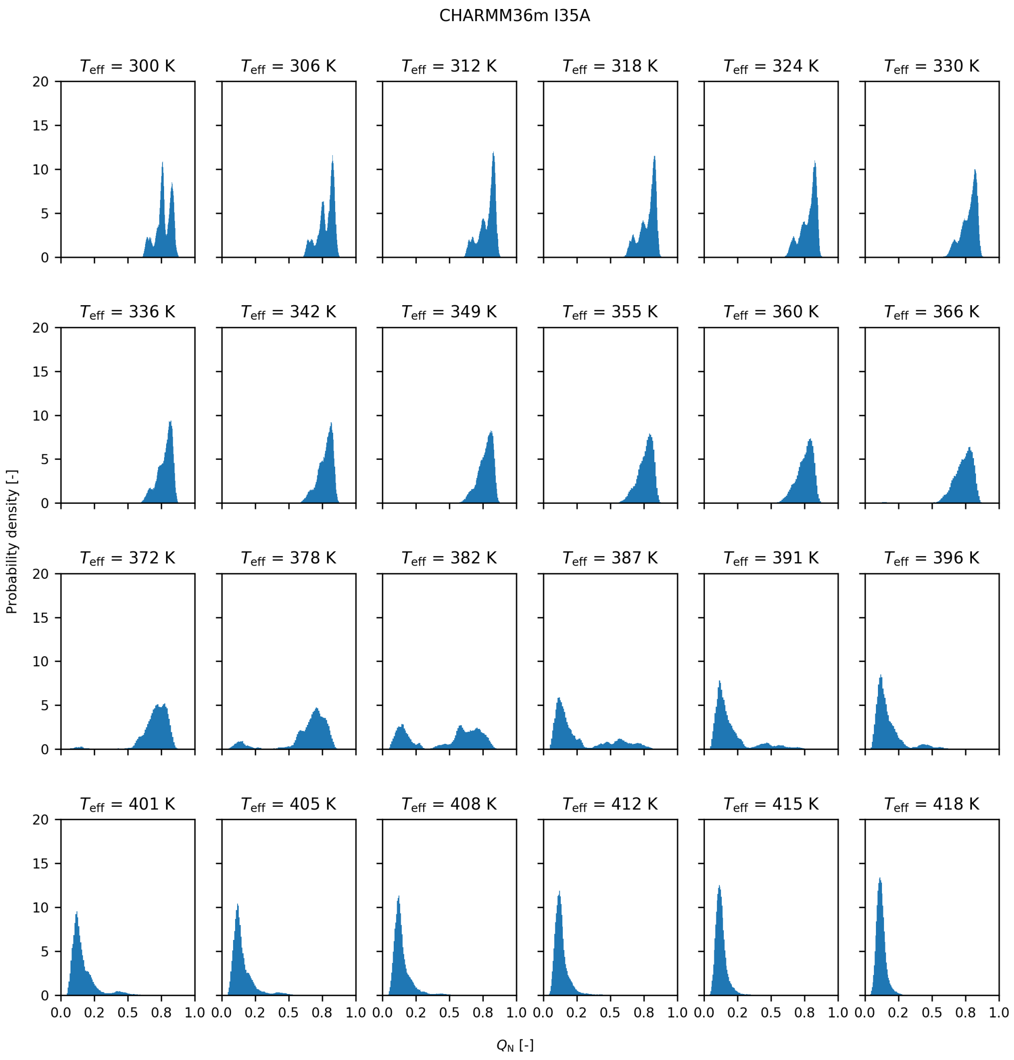

Figure S5: Temperature-dependent distributions of the fraction Q_{N} of $\mathrm{SOD1}_{\mathrm{bar}}^{\mathrm{I35A}}$ native contacts sampled with CHARMM36m.

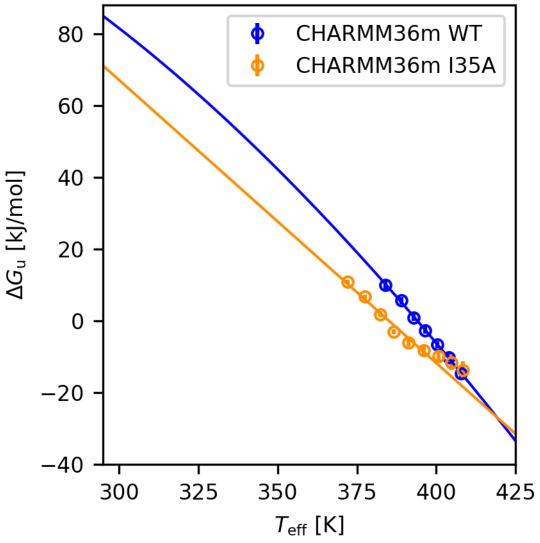

Figure S6: Stability curves obtained from a two-state model for the CHARMM36m REST2 simulations. The lines represent fits using the modified Gibbs-Helmholtz equation (see Supplementary Methods for details).

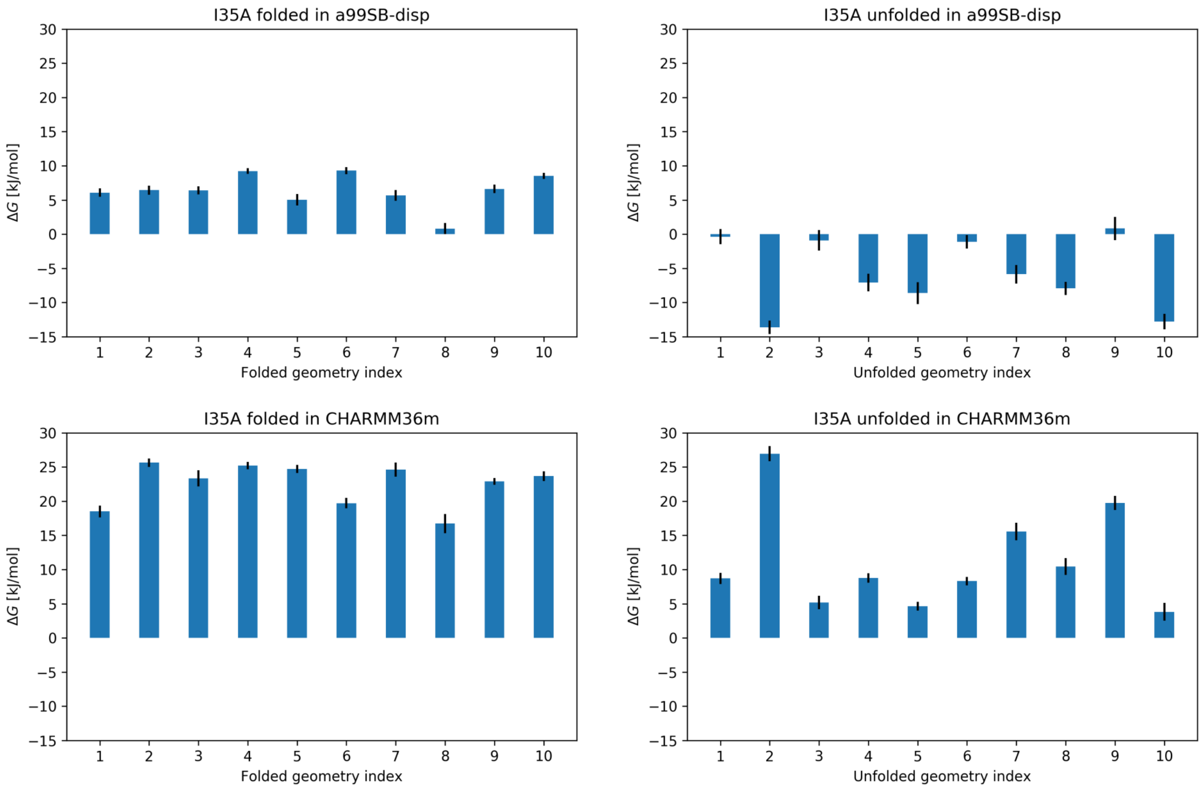

Figure S7: Free-energy difference arising from the alchemical transformation of isoleucine 35 to alanine for individual geometries extracted from the $SOD1^{WT}_{bar}$ REST2 simulations.

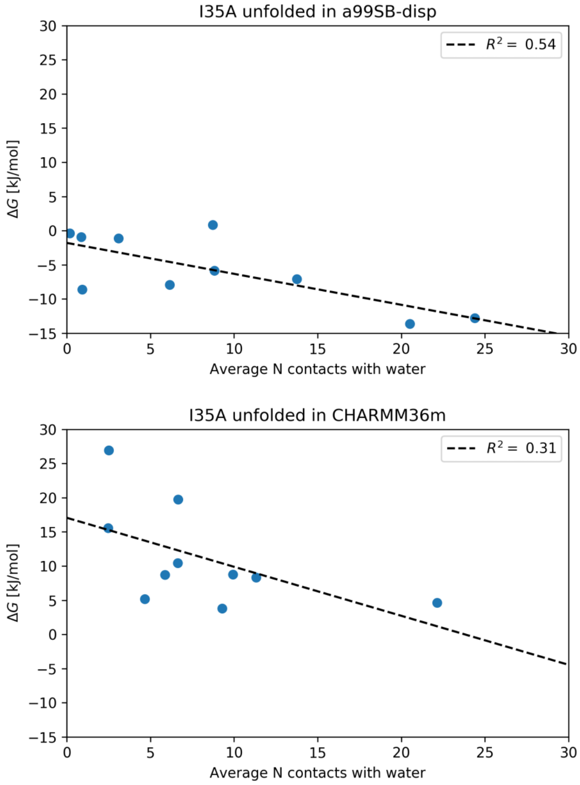

Figure S8: Correlation between the number of water contacts of the I35 side chain and the free-energy differences arising from the I35A alchemical transformation for individual unfolded geometries.

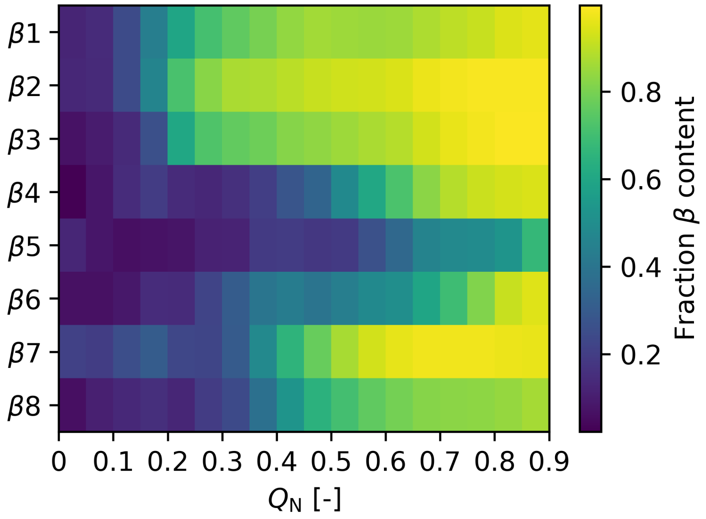

Figure S9: Overall histogram of the β-sheet secondary-structure content in individual β-strands of $\mathrm{SOD1}^{\mathrm{WT}}_{\mathrm{bar}}$ as a function of the native contact fraction Q_{N} and obtained with the CHARMM36m force field. The values are normalized with respect to the crystal structure.

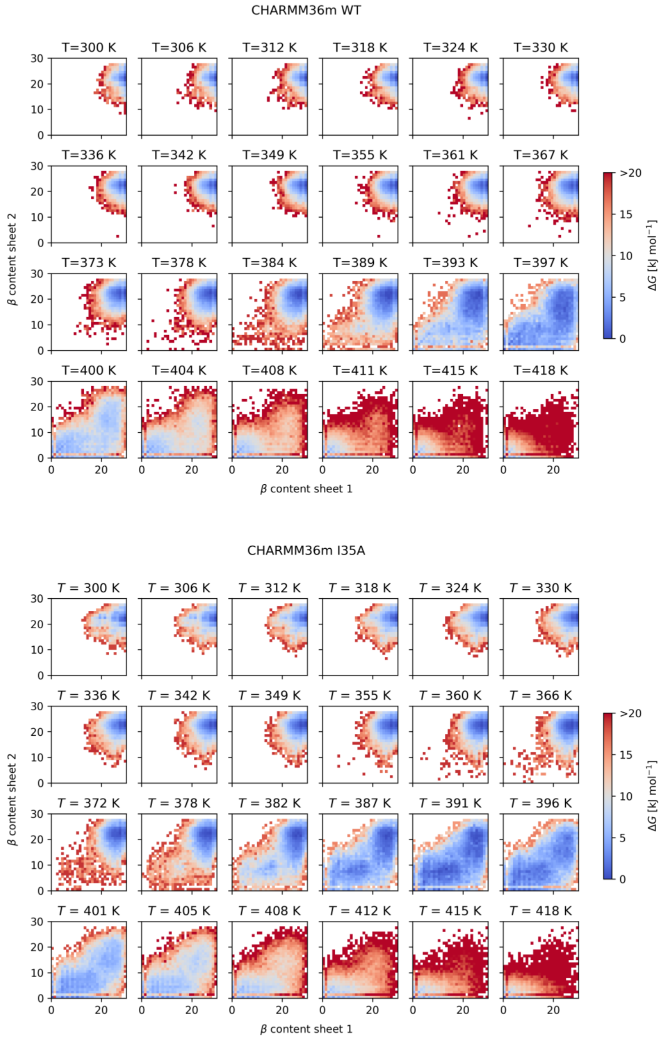

Figure S10: Free-energy landscapes of $\mathrm{SOD1}^{\mathrm{WT}}_{\mathrm{bar}}$ unfolding at different temperatures of the CHARMM36m REST2 simulation. The two collective variables correspond to the number of residues with the β-sheet secondary structure in the β-sheet 1 (formed by the β-strands $\beta 1$, $\beta 2$, $\beta 3$, and $\beta 6$) and the β-sheet 2 ($\beta 4$, $\beta 5$, $\beta 7$, and $\beta 8$).

Supplementary Tables

Step	Length [ns]	Δt [fs]	Restraints	k_{BB} [kJ mol^{-1} nm^{-2}]	k_{SC} [kJ mol^{-1} nm^{-2}]
1	0.1	1	heavy atoms	1000	500
2	0.1	1	heavy atoms	500	250
3	0.1	1	heavy atoms	250	100
4	0.5	2	heavy atoms	100	50
5	0.5	2	heavy atoms	50	0
6	0.5	2	none	0	0

Table S1: Relaxation protocol used to obtain the initial protein geometry. k_{BB} and k_{SC} – force constants of harmonic restraint potentials applied to the heavy atoms of the backbone and the side chains, respectively. Δt – time step.

T_0	300	T_{12}	465
T_1	311	T_{13}	482
T_2	323	T_{14}	500
T_3	335	T_{15}	519
T_4	347	T_{16}	538
T_5	360	T_{17}	558
T_6	373	T_{18}	579
T_8	387	T_{19}	600
T_8	402	T_{20}	623
T_9	417	T_{21}	647
T_{10}	432	T_{22}	672
T_{11}	448	T_{23}	698

Table S2: Solute temperatures [K] used in the REST2 simulations.
